# Clinical recall from the NHS Breast Screening Programme: is it worth doing?

**DOI:** 10.1186/bcr3289

**Published:** 2012-11-09

**Authors:** M Morgan, J Helsdon, A Thomson

**Affiliations:** 1 Dorset Breast Screening Unit, Poole, UK

## Introduction

Within the NHSBSP, ladies with normal mammograms may be recalled, if they proffer significant clinical symptoms. A clinical recall may then be initiated by the reader according to local guidelines. The purpose of this study was to determine the recall rate of ladies undergoing clinical recall and the cancer detection rate in this group.

## Methods

The entire South West and South Central Health Authority screening population was examined for 2011. The study analysed the number of ladies undergoing breast screening, the number of ladies proceeding to clinical recall and the number of cancers detected following clinical recall.

## Results

A total of 342,628 women were screened within these two regions in 2011. Of these ladies, 884 were recalled for a clinical recall. This represents an overall clinical recall rate of 0.26% or one clinical recall for every 388 patients screened. Of the 884 patients recalled, 17 of these patients were subsequently diagnosed with breast cancer. This represents one breast cancer detected for every 52 women clinically recalled to assessment.

## Conclusion

Clinical recall does detect breast cancer but 52 women must be recalled to detect one cancer. These ladies are included in the recall rate of the NHSBSP, but the cancer detection rate in this subset of patients is relatively poor compared with those recalled with abnormal mammography. The NHSBSP should evaluate whether this is an acceptable detection rate or use of resources. An alternative is that these ladies could be directed to their GP, and referred onwards to the symptomatic breast service, when clinically appropriate.

